# Parkinson’s Disease: No Milk Today?

**DOI:** 10.3389/fneur.2014.00172

**Published:** 2014-09-05

**Authors:** Andrea Kistner, Paul Krack

**Affiliations:** ^1^Movement Disorder Unit, Department of Psychiatry and Neurology, University Hospital Grenoble, Grenoble, France; ^2^Institut national de la santé et de la recherche médicale, Unité 836, Equipe 11, Grenoble Institut des Neurosciences, Grenoble, France; ^3^Joseph Fourier University, Grenoble, France

**Keywords:** Parkinson’s disease, diet, milk, dairy products, risk factors, eating behavior

## Abstract

Several prospective epidemiological studies on large cohorts have consistently reported an association between milk intake and a higher incidence of Parkinson’s disease (PD). Pesticide contamination of milk and milk’s urate-lowering effects have been put forward as risk factors to explain epidemiological data. This has led to considerable uncertainty among physicians and avoidance of dairy products by PD patients. However, neither factor stands up to the rational and detailed examination of the literature carried out in this mini-review. We suggest that changes in eating behavior related to pre-motor PD are an alternative potential explanation of correlations observed between milk intake and PD occurrence. Despite clear-cut associations between milk intake and PD incidence, there is no rational explanation for milk being a risk factor for PD. Based on current knowledge, limiting the consumption of dairy products does not seem to be a reasonable strategy in the prevention of the development and progression of PD.

## Introduction

Parkinson’s disease (PD) is a neurodegenerative disease, characterized by a progressive loss of dopaminergic neurons originating in the *Substantia nigra pars compacta*. The etiology is unknown, and environmental factors are thought to play a role in neurotoxicity, especially factors contributing to oxidative stress, such as pesticides or heavy metals (1). Dietary factors may be relevant as they can alter the redox balance in the brain or serve as a vehicle for environmental neurotoxins. Data from prospective epidemiological research has made the study of any link between PD and dietary factors possible. One such finding, recently recapitulated in a meta-analysis (2), was a positive association between milk intake and PD risk. Due to speculation and warnings in the lay press, on the internet and from physicians, many PD patients tend to avoid dairy products. This precaution may worsen the quality of their diet, especially that of elderly patients, who are at risk of under-nutrition, loss of weight and muscle mass (3), osteoporosis (4), and hip fracture (5).

## Dairy Products in Nutrition

Most milk products consumed in human nutrition come from cow’s milk. The significance of milk in human nutrition is related to its content in protein (3%), fat (3–4%), carbohydrate (lactose) (4–5%), calcium (1200 mg/L), and other minerals, microelements, and vitamins (6). The protein fraction is of high quality in terms of digestibility and amino acid composition (6) and encourages skeletal muscle growth (7), which contributes to the prevention of sarcopenia, especially in the elderly.

Bioactive peptides resulting from enzymatic hydrolysis of milk proteins exert multiple biological – such as immunomodulatory and antihypertensive – actions, and are thought to play a protective role in human health (6). Milk and dairy products are the most important sources of calcium in human nutrition and their consumption is related to higher bone density, which may contribute to osteoporosis prevention (6, 8). Consumption of dairy products is also associated with improved metabolic health, and epidemiological evidence points to the contribution of milk in the prevention of metabolic disorders such as excess weight, type 2 diabetes, and cardiovascular disease (7).

Global recommended adult dietary calcium allowances range from 700 to 1300 mg/day (7) and milk products are a good source of calcium with high bioavailability. Because of their nutritional values, dairy products are highly recommended, especially for older people (6–8) and special recommendations also exist for ethnic groups with a high prevalence of lactose intolerance (9).

## Dairy Products and PD Risk: Epidemiological Data

A positive association between milk consumption and PD risk has been found in large prospective cohort studies in the United States, Finland and Greece (10–14) (Table 1). A recent meta-analysis reported a combined risk of 1.4 for highest vs. lowest levels of dairy food intake, with a stronger association for men than for women (2).

**Table 1:** **Consumption of dairy products and incidence of PD**.

Reference	Study cohort	origin	*n*/PD cases	Follow-up (years)	Findings
Chen et al. (14)	HPFS^a^ + NHS^a^	USA	135894/394	18–22	Positive association: milk and PD (men > women)
Park et al. (11)	Honolulu Heart Program	USA/J	7504/128	30	Positive association milk and PD (men) but no association with Calcium, total fat, protein, cheese, butter, ice-cream
Chen et al. (13)	CPS^a^ II Nutrition cohort	USA	130867/388	9	Positive association: dairy products and PD (men > women), milk > dairy products
Kyrozis et al. (10)	Prospective cohort (EPIC^a^)	Greece	26173/120	8	Positive association milk, but no association with cheese and yogurt
Sääksjärvi et al. (12)	Finnish Mobile Clinic Survey	Finland	4524/85	41	Milk (women)

*^a^NHS, National Health Study; HPFS, health professional follow-up study; EPIC, European Prospective Investigation into Cancer and Nutrition; CPS, Cancer Prevention Study*.

Overall, the association between PD and milk was stronger than the association with other dairy products such as cheese, yogurt, or butter (2). We do not know which of the components of milk mediates this effect. It is unlikely to be due to milk compounds such as calcium, vitamin D, total fat, or total protein as these compounds are not associated with PD when derived from other sources (11, 14). It was hypothesized that low levels of pesticides or other neurotoxic chemicals may be present in milk and could accumulate in the brain and increase PD risk (15). However, this hypothesis is rather unlikely as skimmed products containing less fat, and therefore a decreased capacity for harboring pesticides, have the same effect (12). Meta-analysis of existing studies (2) has shown that the link between butter and the risk of PD is even lower. Park et al. speculated that genetic characteristics, such as lactose intolerance could be protective. However, the association between milk intake and PD persisted after men who consumed no milk whatsoever (presumably those with lactose intolerance) were excluded (11).

Another theory points to a milk-induced shift in the metabolic redox balance: it was shown that ingestion of milk can decrease serum urate levels in healthy volunteers (16). Urate is a potent endogenous antioxidant, which can inactivate potentially harmful neurotoxic substances, such as free radicals or iron (17). A recent meta-analysis confirmed low urate levels in PD, a finding, which could point to a neuro-protective role of urate (18).

However, we lack evidence of either (a) a role of low urate levels in neuro-degeneration or (b) an association between milk and metabolic antioxidative defense.

## Consumption of Dairy Products in Pre-Motor PD: A Result of Altered Eating Behavior?

We hypothesize that psychological and emotional alteration in pre-motor PD may induce modifications in eating behavior. Non-motor symptoms, such as depression, anxiety, or apathy may be present in PD and precede motor symptoms (19). Associations between foods and moods, especially depressive symptoms, have been reported (20, 21). But why would these patients prefer milk to all other foods? One possible hypothesis is that milk consumption could be associated with mood alterations, as has been shown in an Australian epidemiologic study, where consumption of dairy products was related to the presence of depressive symptoms (22). This could be linked to alpha-lactalbumin, one of the main protein components of milk, which is rich in tryptophan, the precursor of serotonin. Serotonergic hypofunction has been described in early PD and may be involved in depression and anxiety (23). A significant rise in cortex tryptophan closely followed by an increase of serotonin synthesis occurred in rats following acute alpha-lactalbumin consumption (24) and this effect persisted chronically (25). In human volunteers an alpha-lactalbumin-enriched diet increases the ratio of tryptophan to the other large neutral amino acids, which is considered to be an indirect indication of increased brain serotonin function (26). In stress-vulnerable individuals, alpha-lactalbumin improved mood and attenuated the cortisol response following experimental stress (26). In individuals with high-trait anxiety, the administration of alpha-lactalbumin had an effect on food hedonics (27). Milk consumption in pre-motor PD may therefore be interpreted as spontaneous self-medication aimed at improving mood. Since the effect would be very discreet, it would only be observed in large-scale epidemiological studies. This hypothesis might also explain why milk is more strongly linked to PD than cheese: In the cheese-making process, the liquid whey containing alpha-lactalbumin is separated from the curd, and cheese does not contain alpha-lactalbumin.

Furthermore, in many Western countries, milk is traditionally considered as a sleep-inducing beverage and could be consumed – because of its content in melatonin or just on belief – by people suffering from sleep disorders (28), which are frequent in pre-motor PD (29).

However, as we have no strong evidence of the mood- or sleep-modifying effects of milk to date, other mechanisms should be considered to explain the epidemiological findings. Other non-motor symptoms that are frequent in pre-motor PD, such as hyposmia or constipation (30), for instance, may alter dietary habits. Dairy foods, especially fermented products, may relieve constipation, or constipated people may think they do. Another hypothesis is increased consumption of dairy products secondary to decreased consumption of other drinks. In fact, coffee consumption was reported to be low in pre-motor PD (31) and may have been replaced by dairy products. Finally, dopaminergic and homeostatic control of eating behavior may be disturbed in pre-motor PD (3), leading to alterations in dietary habits which may explain the findings.

## Conclusion

To date, the explanation for the epidemiological link between dairy products and PD remains unknown. Evidence that milk constitutes a risk factor in PD is lacking. Limitation of milk consumption cannot therefore be recommended, just as smoking or coffee drinking cannot be recommended in order to prevent PD. Foods that supply calcium and high-quality protein should not be limited in view of the high prevalence of osteoporosis and hip fracture in PD (4, 5). Because of their high nutritional qualities, consumption of milk and dairy products should be encouraged in PD to the same degree as in the general population. Interference with dopaminergic treatment may be canceled out by respecting appropriate time lags between medication intake and meals. In the case of lactose intolerance, fermented dairy products, such as yogurt or cheese, may replace milk.

## Conflict of Interest Statement

The authors declare that the research was conducted in the absence of any commercial or financial relationships that could be construed as a potential conflict of interest.
